# Heterogeneous focal adhesion cytoskeleton nanoarchitectures from microengineered interfacial curvature to oversee nuclear remodeling and mechanotransduction of mesenchymal stem cells

**DOI:** 10.1186/s11658-025-00692-z

**Published:** 2025-01-24

**Authors:** Huayu Fan, Hui Zhao, Yan Hou, Danni Meng, Jizong Jiang, Eon-Bee Lee, Yinzheng Fu, Xiangdong Zhang, Rui Chen, Yongtao Wang

**Affiliations:** 1 Luoyang Orthopedic-Traumatological Hospital Of Henan Province (Henan Provincial Orthopedic Hospital), Zhengzhou, 450008 Henan China; 2 Zhengzhou Revogene Technology Co., LTD, Airport District, Zhengzhou, 451162 Henan China; 3 School of Medicine, Shanghai University, Shanghai, 200444 China; 4 Department of Aquatic Life Medicine, Pukyong National University, Busan, 48513 Republic of Korea; 5 School of Nursing, Fujian University of Traditional Chinese Medicine, Fuzhou, 350122 Fujian China

**Keywords:** Cell colony, Interfacial heterogeneity, Focal adhesion, Cytoskeleton tension, Nuclear mechanotransduction

## Abstract

**Background:**

Interfacial heterogeneity is widely explored to reveal molecular mechanisms of force-mediated pathways due to biased tension. However, the influence of cell density,, curvature, and interfacial heterogeneity on underlying pathways of mechanotransduction is obscure.

**Methods:**

Polydimethylsiloxane (PDMS)-based stencils were micropatterned to prepare the micropores for cell culture. The colonies of human mesenchymal stem cells (hMSCs) were formed by controlling cell seeding density to investigate the influences of cell density, curvature and heterogeneity on mechanotransduction. Immunofluorescent staining of integrin, vinculin, and talin-1 was conducted to evaluate adhesion-related expression levels. Then, immunofluorescent staining of actin, actinin, and myosin was performed to detect cytoskeleton distribution, especially at the periphery. Nuclear force-sensing mechanotransduction was explained by yes-associated protein (YAP) and laminA/C analysis.

**Results:**

The micropatterned colony of hMSCs demonstrated the coincident characters with engineered micropores of microstencils. The cell colony obviously developed the heterogeneous morphogenesis. Heterogeneous focal adhesion guided the development of actin, actinin, and myosin together to regulate cellular contractility and movement by integrin, vinculin, and talin-1. Cytoskeletal staining showed that actin, actinin, and myosin fibers were reorganized at the periphery of microstencils. YAP nuclear translocation and laminA/C nuclear remodeling were enhanced at the periphery by the regulation of heterogeneous focal adhesion (FA) and cytoskeleton arrangement.

**Conclusions:**

The characters of the engineered clustering colony showed similar results with prepared microstencils, and colony curvature was also well adjusted to establish heterogeneous balance at the periphery of cell colony. The mechanism of curvature, spreading, and elongation was also investigated to disclose the compliance of FA and cytoskeleton along with curvature microarrays for increased nuclear force-sensing mechanotransduction. The results may provide helpful information for understanding interfacial heterogeneity and nuclear mechanotransduction of stem cells.

**Supplementary Information:**

The online version contains supplementary material available at 10.1186/s11658-025-00692-z.

## Introduction

Interfacial heterogeneity is an essential consideration for administering cell functions and fates in various organisms [1–3]. The encapsulation of cells is mainly divided into two aspects. One is the neighboring cell–cell touch, and another is the surrounding microenvironment of cell survival, or extracellular matrix (ECM) [4]. The biological, chemical, and physical stimulations are perceived from both conditions to excite downstream force-related signal pathways, such as Wingless-related integration site (Wnt) and Hippo activation [5, 6]. Adjacent cells can rely on the formation of molecular proteins of cadherin, tight junction, and desmosome in contact with each other on the cell membrane to transmit mechanical signals between them [7]. Besides cell–cell communication, extracellular heterogeneous microenvironment and ECM also serve as the decisive components in governing cell behaviors, including cell morphogenesis, migratory ability, and destiny evolution [8–12]. Microenvironment-mediated biophysical responses induce the secretion of adhesion spots, recombination of cytoskeleton structure, and changes in intracellular osmotic pressure homeostasis, which affect the heterogeneous mechanics of cells [13, 14]. Compared with single cell culture, multiple-cell clustering on the bioengineered surfaces can better mimic complex conditions to investigate cytoskeletal organization and nuclear DNA expression levels [15, 16]. Further, cancer cells can exhibit heterogeneous interfaces at the periphery of engineered cell clusters, which allows them to express unique phenotypes [17–20]. Moreover, the morphogenesis and tumorigenesis of cancer stem cells are also regulated by altering the interfacial heterogeneity of geometry on micropatterns [21]. The cells are cultured on the micropatterns to change their morphogenesis and interact with the curvature interface to promote more focal adhesion (FA) and cytoskeleton formation [22]. These force-sensing proteins can transfer extracellular stimuli by transmembrane adhesion proteins (vinculin or paxillin) into cells to effect cytoskeleton reorganization [23]. Heterogeneous curvature at the adhesive periphery could also enhance adhesion protein expression by activating mechanosensing through the regulation of yes-associated protein (YAP) nuclear localization and laminA/C nuclear remodeling [24]. Cell composition and its interaction with the heterogeneous curvature indicate that cell adhesion is reinforced by interfacial heterogeneity to improve cell force-sensing behaviors and nuclear mechanotransduction [25].

When cells are attached to a heterogeneous microenvironment, especially on different matrix surfaces, they can show a variety of morphological characteristics, which alter the mechanical state of cells and transmit heterogeneous mechanical signals into cells to respond to these stimuli. This is crucial to maintain cell activity and tissue metabolism [26–28]. Recent studies have found that cells in biological tissues have different densities and shapes to adapt to the alteration of the extracellular microenvironment, and cell micromorphology, density, size, and chirality exhibit typical features that regulate cell functions [29–31]. Meanwhile, cell heterogeneity has a vital function in energy-mediated cell internalization, and it can adjust the intracellular gene phagocytosis and transfection [32, 33]. In addition, heterogeneous cell biomechanics are of great significance to the repair of soft and hard tissues in vivo, especially the regenerative repair performance of various tissues and organs, such as bone, cartilage, fat, the cardiovascular system, and nerves [34–37]. Cell biomechanical behaviors can also guide the diversity of internal mechanical signals, thus accelerating the ability to repair tissue [38]. Although heterogeneous architecture is widely studied in disclosing the molecular mechanism of mechanical pathways, the influence of curvature-dependent interfacial heterogeneity on nuclear mechanotransduction remains unclear in mesenchymal stem cells (MSCs). Herein, polydimethylsiloxane (PDMS)-based stencils were micropatterned to assist the formation of heterogeneous cell colonies by controlling colony curvature and cell seeding density. The heterogeneity of FA proteins (integrin, vinculin, and talin-1) and cytoskeleton filaments (actin, actinin, and myosin) was explored to reveal nuclear mechanotransduction by the evaluation of YAP nuclear translocation and laminA/C remodeling. The study will provide valuable information for interfacial heterogeneity and mechanotransduction of stem cells.

## Material and methods

### Micropatterning and analysis of PDMS-based stencils

PDMS-based microstencils were micropatterned via a simple punching technology [31]. Briefly, PDMS-based films, 100 μm thick, were microengineered using a punching method by the Chinese Hangzhou Bald Company. In the beginning, 100-μm PDMS-based films were punched into 1.4-cm-diameter circles through an engineered scissor. The PDMS films were put in 75% alcohol for 0.5 h to eliminate residual PDMS monomer and washed with phosphate-buffered saline (PBS). After that, a specific puncher, which was composed of 800-μm and 1500-μm inner diameters, was applied to perforate PDMS-based films to form the through-film stencils, and two-type microstencils were considered as D-800 and D-1500, respectively. For sterilization, the punched PDMS-based microstencils were immersed in 75% alcohol for 0.5 h and washed with PBS three times. Finally, PDMS-based microstencils were put into 24 well dishes for hMSC assays.

### Culture of hMSCs

Human mesenchymal stem cells (hMSCs, 2F3478) were obtained from American Lonza Walkersville Inc. The cells were cultured in a Dulbecco’s modified eagle medium (DMEM) culture medium. The DMEM medium was enriched with 10% fetal bovine serum (FBS) and 1% penicillin-streptomycin (PS). After 80% confluence of hMSCs, the cells were harvested using ethylenediamine tetraacetic acid (EDTA)/trypsin treatment and centrifuged at 300 g. The cells were suspended in the culture medium to build homogenous hMSC suspension with cell densities of 0.5 × 10^5^, 1.0 × 10^5^, and 2.0 × 10^5^ cells mL^−1^. PDMS stencils with a diameter of 14 mm were tightly adhered to the tissue-culture-treated 24-well polystyrene plates (FCP243, Beyotime Biotechnology, China). A total of 1 mL cell suspension was put into the PDMS stencils to adjust the seeding density of hMSCs, and the three seeding conditions of hMSCs were considered as low-level, middle-level, and high-level density. After a 6 h culture of hMSCs, the DMEM was refreshed with a new culture medium, and the hMSCs were grown in microstencils for 18 h to form the desired cell density, geometrical morphogenesis, and spatial heterogeneity.

### Regulation of nuclear staining on cell distribution

When the hMSCs were seeded on the microstencils for 1 day, the PDMS-based stencils were peeled off to exhibit the microcolony of hMSCs. The cells were washed with PBS three times and fixed with 4% paraformaldehyde (PFA). The hMSCs were handled with 1% Triton X-100 and the nuclei were stained with 4’,6-diamidino-2-phenylindole (DAPI, Shandong Sparkjade Company, China). The stained fluorescence of hMSCs was captured via a fluorescent microscope (MF52-N Guangzhou Micro-Shot Company, China). The captured images were applied to calculate the density of hMSCs in each microcolony. The density of hMSCs was calculated according to various seeding densities on the basis of the fluorescent images. Each cell colony was also distinguished between the central area and peripheral area. Assuming that the central area was defined as equal space with the peripheral area, the length of microring at the periphery would be analyzed as 117 μm for 800-μm stencils and 219 μm for 1500-μm stencils. The amount of hMSCs at different areas was calculated to regulate the peripheral and central density of hMSCs by ImageJ, and five independent experiments were performed to analyze the mean and standard deviation (SD).

### Focal adhesion analysis by integrin, vinculin, and talin-1 immunofluorescent staining

The production of focal adhesion (FA) was evaluated in different areas of the MSC colony by vinculin. The hMSCs were seeded on the microstencils for 1 day; the PDMS stencils were directly removed to display the microcolony and washed with PBS. The cells were fixed with 4% cold PFA and handled with Triton X-100 and Tween-20. Then, 2% BSA was applied to block the cells. For vinculin staining, vinculin primary antibody (Merck, Germany) was applied to incubate the cells. After the hMSCs were washed with PBS three times, Alexa Fluor 488-labeled immunoglobulin (Ig)G antibody (Sigma-Aldrich, USA) was applied to stain the cells in the dark. Then, DAPI was applied to stain the nuclei. Fluorescent pictures were taken via a fluorescent microscope to analyze the FA formation of stained hMSCs. The average size of vinculin was regulated according to the fluorescent images using a step-by-step method via ImageJ, as previously reported [28]. A total of five independent experiments were performed to analyze the mean and SD. For integrin and talin-1 staining, the cells were incubated with integrin αV primary antibody (Cell Signaling Technology, USA) and talin-1 primary antibody (Cell Signaling Technology, USA) overnight, respectively. Alexa Fluor 488-labeled IgG antibody was applied to stain the cells in the dark for 1 h to regulate integrin and talin-1 formation through a fluorescence microscope.

### Actin fluorescent staining and immunofluorescent treatment of actinin and myosin

After 1 day of culture, the hMSCs were followed by three PBS washes and fixed with 4% PFA. Then, the fixed hMSCs were treated with Triton X-100 and blocked with 2% BSA. Nuclei and actin were costained with 1‰ DAPI and 2% Alexa Fluor-594 phalloidin in the dark, respectively. The fluorescent images of hMSCs were observed through the fluorescent microscope. The cellular aspect ratio was calculated on the basis of actin organization by ImageJ. A total of five independent experiments were conducted to analyze the mean and SD.

For immunofluorescent staining of actinin and myosin, the hMSCs were treated with their respective primary antibody, 1% actinin primary antibody (Abcam, USA) for actinin and 1% myosin primary antibody (Sigma-Aldrich) for myosin. After the hMSCs were washed with PBS, Alexa Fluor 488-labeled anti-mouse IgG antibody was applied to stain actinin and Alexa Fluor 488-labeled anti-rabbit IgG antibody (Sigma-Aldrich) for myosin. The fluorescence pictures of hMSCs were observed through a fluorescent microscope. In addition, the heatmap images (> 20 images) of myosin were superposed along with the *z*-axis to evaluate the myosin distribution at peripheral and central areas.

### Regulation of mechanotransduction by YAP staining

Mechanotransduction of hMSCs was analyzed by YAP (Thermo Fisher Scientific, USA) staining. After 1 day of culture, the hMSCs were fixed with 4% cold PFA for 10 min, treated with 1% Triton X-100 for 10 min, and blocked with 2% BSA for 0.5 h. After that, mouse anti-YAP primary antibody (1:200 in BSA, Santa Cruz Biotechnology, USA) was applied to incubate the cells overnight and washed with PBS three times. Fluorescent secondary antibody was carried out with Alexa Fluor-488 labeled IgG antibody. DAPI was applied to stain nuclei. Fluorescence pictures were observed by a fluorescent microscope. YAP nuclear localization was evaluated by calculating the percentage of YAP localized in the nucleus of all assessed hMSCs. A total of five independent assays were performed to analyze the mean and SD.

### Preparation of engineered microcircles and BrdU staining

Photosensitive polymer (PVA) was synthesized, as previously reported [16]. PVA solution was coated on cell culture dishes to form a nano-scale layer. A photomask was covered on the dishes to expose ultraviolet (UV) light to produce the microcircles. After washing, the microstructures were formed on culture dishes. The cells were seeded on the microengineered surfaces to control cell morphology. After 1 day of culture, the cells were fixed and stained with a BrdU primary antibody (1:200 in BSA) overnight and the Alexa Fluor-488 labeled IgG antibody (1:1000 in BSA) for 1 h. BrdU positive nuclei were summed to calculate nuclear DNA synthesis activity. A total of five independent assays were performed to analyze the mean and SD.

### Western blot (WB)

The cells were collected in low- and high-density conditions after 24 h incubation. WB analysis was used to detect the expression of related proteins (integrin, vinculin, YAP, and laminA/C). The utilized primary antibodies were integrin αV primary antibody (Cell Signaling Technology, USA), laminA/C primary antibody (Cell Signaling Technology, USA), and vinculin primary antibody (Merck, Germany). In addition, the cells were treated with 50 μM blebbistatin to disturb myosin assembly for 6 h to assess the regulation of integrin, vinculin, and laminA/C nuclear expression levels by WB analysis. The typical stencils were engineered by punching method for each type stencil.

### Statistical evaluation

Statistical evaluation was applied to analyze the quantitative fluorescence pictures. In this work, the data are presented as the mean ± SD. The difference was analyzed using analysis of variance (ANOVA). When *p* < 0.05, it was considered to be a significant difference.

## Results and discussion

### Characterization of bioengineered microstencils and colony culture of hMSCs

**Fig. 1:**
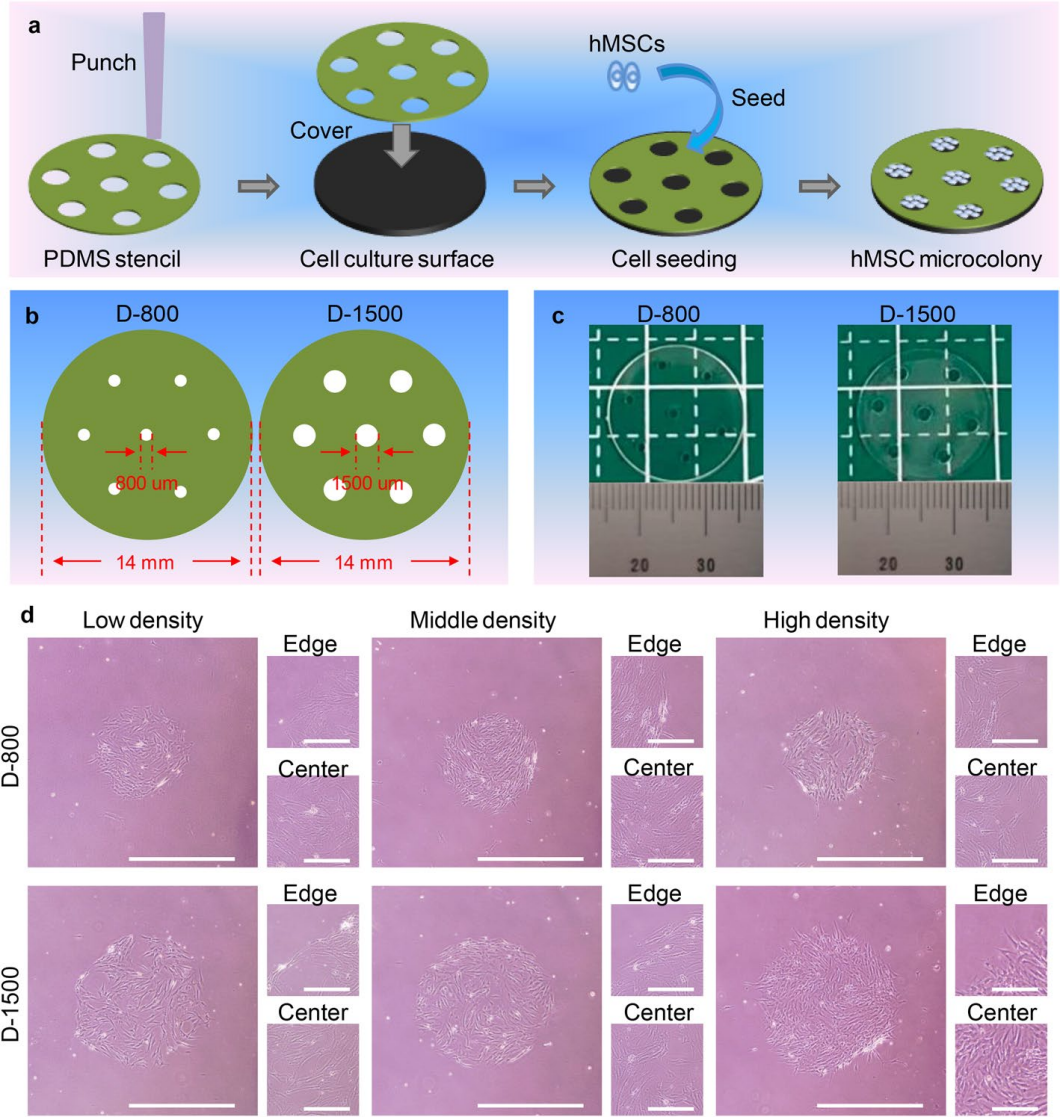
Characteristics of polydimethylsiloxane (PDMS) stencils and the characterization of cell colony of human mesenchymal stem cells (hMSCs). **a** Illustration of PDMS-based microstencils preparation and cell seeding; **b** geometrical features of microstencils, two-type microstencils were considered as D-800 and D-1500 for the diameters of 800 and 1500 μm; **c** digital pictures of PDMS-based microstencils, a millimeter ruler is shown at the bottom; **d** microscopic pictures of a clustering colony of hMSCs. The scale bar represents 1000 μm. Right two pictures for each density are the amplifications at both central and edge areas. The scale bar represents 100 μm

The biofunctional microstencil method has been reported to regulate cell colony formation, especially for geometrical morphogenesis and spatial heterogeneity [39]. To investigate the influence of interfacial heterogeneity on intracellular mechanotransduction of stem cells, PDMS-based microstencils were created utilizing a punching bioengineering method to control cell morphology of hMSCs (Fig. 1a). In particular, the PDMS-based films with a thickness of 100 μm were punched to create film-penetrated micropores via commercial punchers. The sizes of micropores were 800 μm and 1500 μm on the two kinds of PDMS-based microstencils, respectively (Fig. 1b). The micropores on PDMS-based microstencils were certificated through digital photos and the round micropores were constructed on PDMS-based microstencils (Fig. 1c). The characteristics of micropores were calculated to be 811.7 ± 12.0 μm and 1554.0 ± 42.0 μm in size, and the circularity of micropores was analyzed to be 0.99 ± 0.03 and 1.10 ± 0.25, respectively. The performance results indicated that PDMS-based micropores were precisely controlled on microstencils.

Prior to cell culture, the PDMS-based microstencils were immersed in a 75% ethanol solution for sterilization. After that, the sterilized PDMS-based microstencils were carefully placed onto the substrates of cell culture surfaces for hMSC seeding. The hMSC suspension was uniformly dropped onto the microstencils and the colony of hMSCs could be established in PDMS-based micropores. The morphology observation of the hMSCs colony was performed via microscope (Fig. 1d). The round colony of hMSCs was formed on 24-well cell culture plates. The amplified pictures exhibited that the hMSCs were homogeneous arrangement in all directions at the central region, but peripheral hMSCs showed obvious compliance with the microhole periphery of PDMS stencils. Moreover, proving the good controllability of the cell colony, the size of the hMSC colony was also calculated as 816 ± 10 μm and 1612 ± 44 μm, and the circularity was measured as 0.98 ± 0.02 and 0.99 ± 0.04, respectively (Supplementary Fig. 1). The quantitative results indicated that cell clusters presented the same features as the micropores of PDMS-based microstencils.

### Regulation of cell seeding density, curvature, and heterogeneity on cell distribution

**Fig. 2:**
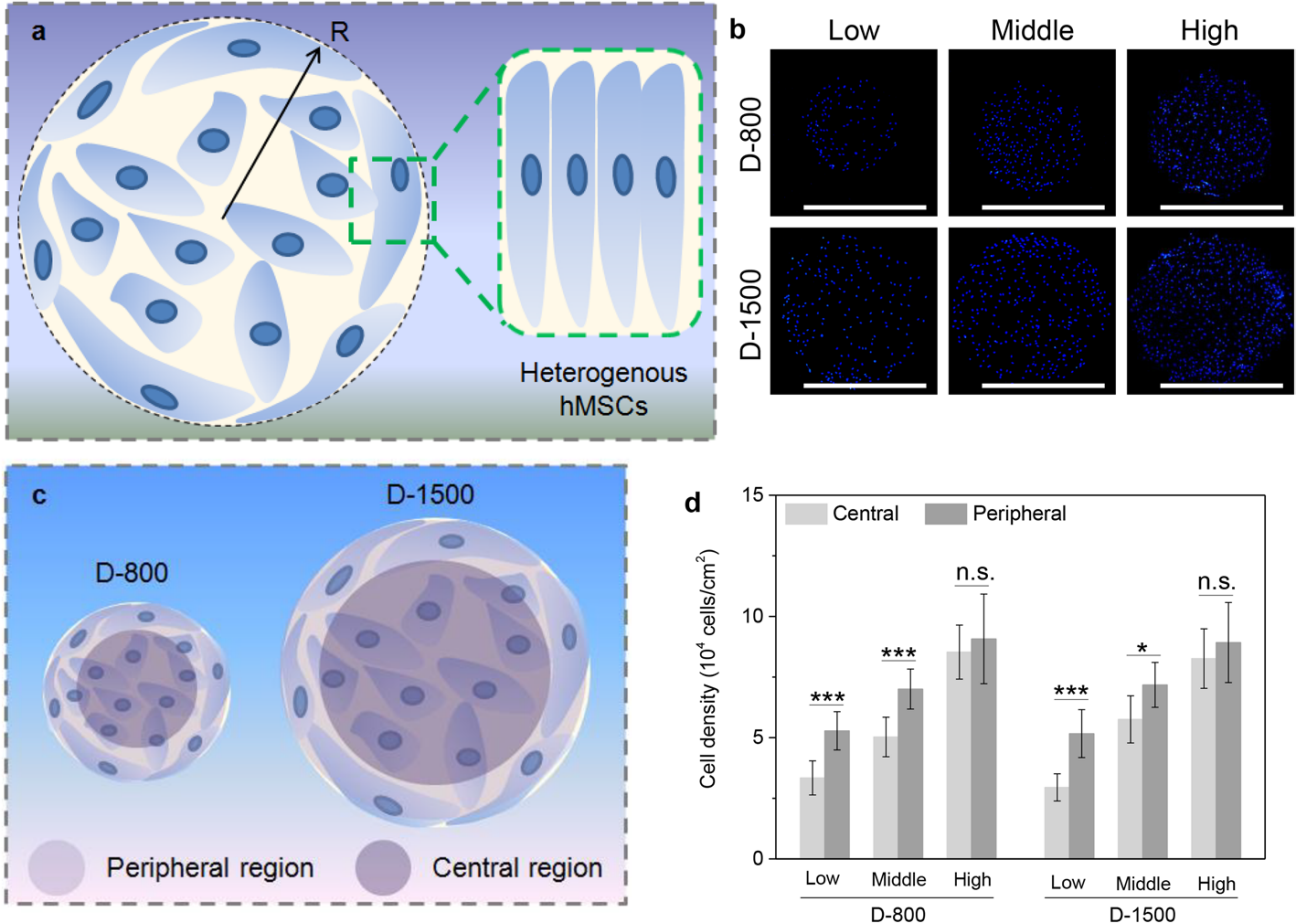
Regulation of cell seeding density, curvature and heterogeneity on cell distribution. **a** Illustration of the compliance of human mesenchymal stem cells (hMSCs) at the periphery; **b** fluorescent pictures of blue 4’,6-diamidino-2-phenylindole (DAPI) nuclei, scale bar represents 1000 μm; **c** definition of central and peripheral areas in the engineered hMSCs; **d** density of hMSCs at both central and peripheral areas of hMSCs in D-800 and D-1500 colonies, data are presented as mean ±  standard deviation (SD), *n* = 5, n.s.: no significance, *p < 0.05, ***p < 0.001

According to cell morphogenesis analysis on PDMS-based microstencils, the hMSCs could change their cluster colony to accommodate the micropores at interfacial regions. The peripheral hMSCs were elongated along with the interfacial microenvironment (Fig. 2a). Therefore, the cells would prefer to adhere to the peripheral areas of micropores. To confirm the hypothesis of interfacial compliance, the microengineered hMSCs were seeded in the micropores of PDMS-based microstencils for 1 day to stain the nuclei blue. This staining allowed for the regulation of the influence of cell density, curvature, and geometrical morphology on cellular distribution and spatial heterogeneity (Fig. 2b). The staining results showed that more hMSCs attached to the peripheral areas in low and middle initial seeding densities, but there was little significant difference in hMSC distribution at central and peripheral areas in high cell seeding density. On the basis of these results, the cells in the peripheral area showed different morphology and density from the cells in the central area. To distinguish the difference, the micropores were divided into two sections of peripheral and central areas (Fig. 2c). As the two regions had the same area to calculate respective cell density distribution, the comparison of cell density was conducted to assess the interplay among cell seeding density, curvature, and heterogeneity (Fig. 2d). Cell seeding density could increase the density distribution of hMSCs at central and peripheral areas. More cells gathered at peripheral regions, while fewer cells assembled at central regions when the seeding density of hMSCs maintained low and middle conditions, independently of the high seeding density of hMSCs. The density data displayed that heterogeneous density distribution was regulated by the seeding density of hMSCs, interfacial curvature, and spatial heterogeneity.

### Evaluation of heterogeneous FAs by engineered cell density and curvature

**Fig. 3:**
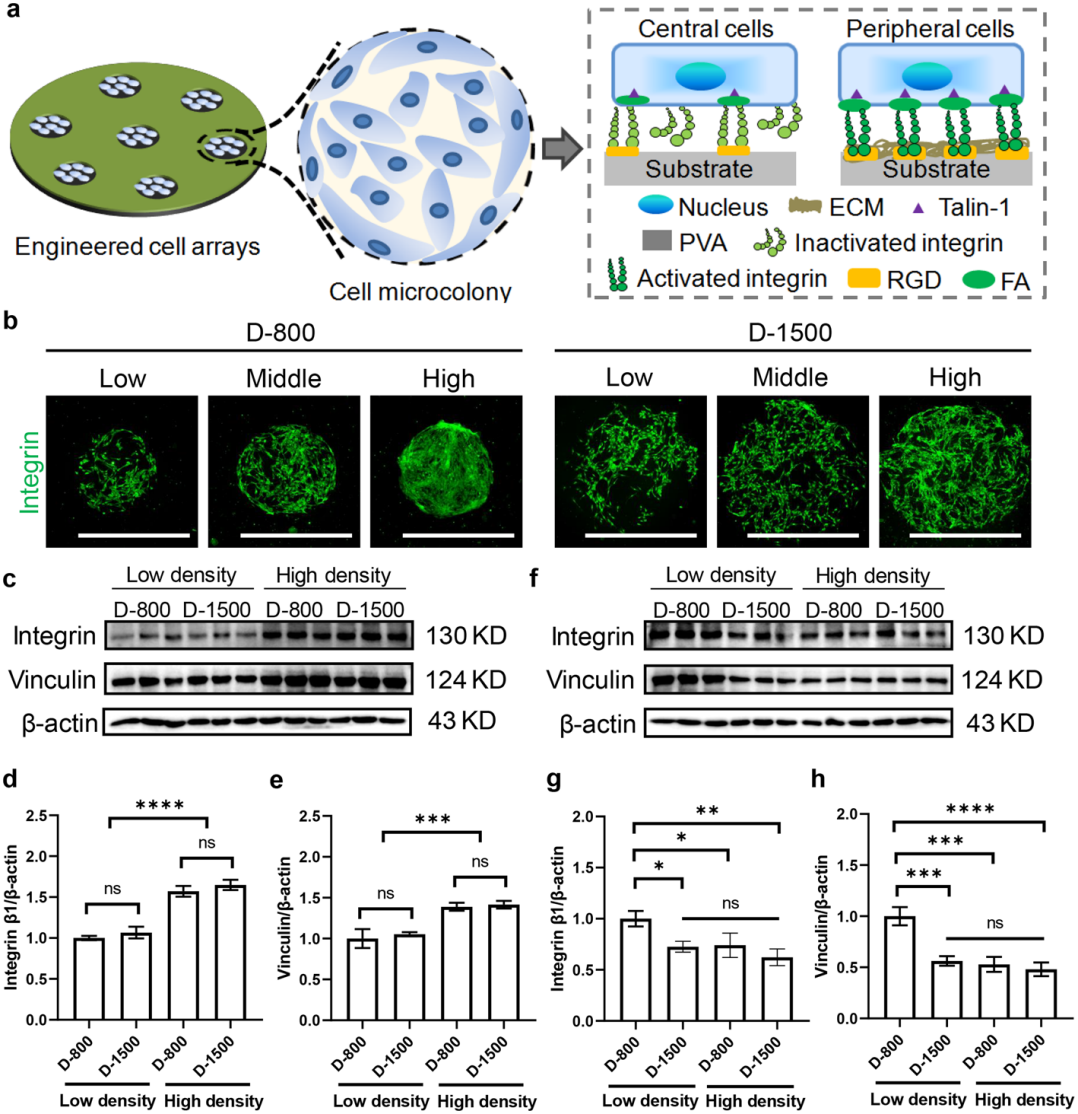
Evaluation of heterogeneous focal adhesions (FAs) by engineered cell density and curvature. **a** Illustration of the engineered FA proteins as a mechanosensor between extracellular matrices (ECMs) and cytoskeletons; **b** fluorescent pictures of integrin proteins (green), scale bar represents 1000 μm; **c** Western blot (WB) analysis of integrin and vinculin in the micropatterned human mesenchymal stem cells (hMSCs) at the central regions; **d** quantitative intergrin/β-actin in central regions; **e** quantitative vinculin/β-actin in central regions; **f** WB analysis of integrin and vinculin in the micropatterned hMSCs at the peripheral regions; **g** quantitative integrin/β-actin in peripheral regions; **h** quantitative vinculin/β-actin in peripheral regions, data are presented as mean ± standard deviation (SD), *n* = 3, ns: no significance, *p < 0.05, **p < 0.01, ***p < 0.001, ****p < 0.0005

Cell-density-induced interaction and crosstalk between the neighboring cells play a vital role in regulating the assembly and formation of adhesion-related cytokines and proteins, such as FAs [40, 41]. The arrangement of FA proteins (integrin, vinculin, and talin-1) can be significantly affected by the cellular detention microenvironment and act as a mechanical sensor to administer cell function and fate [42]. In this study, the hMSCs were seeded in different micropores of PDMS stencils to assemble cellular FA proteins and transmit biomechanical stimuli from the extracellular microenvironment to the hMSCs and their nuclei. On the basis of this assumption, FA proteins probably serve as the bioacceptor of extracellular stimulation between ECMs and cytoskeletons (Fig. 3a). Integrin was stained green to investigate the formation of adhesion-related proteins (Fig. 3b). The staining results suggest that the colony can induce integrin formation in all engineered cells. The quantitative analysis of fluorescent intensity showed that integrin increased with cell density, and the cells in peripheral regions formed more integrin than those in central regions (Supplementary Fig. 2a).

Vinculin was the most important component of FA proteins and was examined by using immunofluorescent staining to explore the interplay of cell density, curvature, and heterogeneity in the arrangement of FA proteins on the microstencils. The nuclei and actin filaments were also costained blue to determine the cell position. The results of vinculin staining displayed that FA proteins were grown and recruited for the entire engineered cell colony (Supplementary Fig. 2b). The total fluorescence of FAs in a complete colony was enhanced by enlarging the cell initial density and pore size. To thoroughly investigate the interplay between interfacial colony (cell density, curvature, and heterogeneity) and FA assembly, the average size of FA proteins was analyzed through a step-by-step method of ImageJ [16]. The cell colony was divided into peripheral and central areas to assess the formation of FA proteins in each part. In specific, in central areas, compared with low and high cell density, middle cell density showed the largest average FA size in both D-800 and D-1500 micropores, independently of D-800 and D-1500 locations (Supplementary Fig. 2c). It may be due to the defect of cell–cell interaction in low cell density and limited nutrient and density-dependent repression in high cell density [43]. Interestingly, the average FA size was changed by cell-density-dependent curvature and heterogeneity in the peripheral areas. Middle cell density at the periphery presented a larger average FA size than low and high cell density. The curvature also altered the average FA size in the peripheral area; strong curvature may promote FA formation in hMSC density with low and middle conditions, but it did not affect FA size in high density of hMSCs. Furthermore, peripheral hMSCs were inclined to assemble larger FA sizes than central cells in micropores of PDMS stencils. In addition, the orientation of focal adhesion was observed, and the results showed that mature focal adhesion could grow along with interfacial curvature of a colony at the periphery, while mature focal adhesion was almost undetected in the central regions.

Further, WB analysis was used to detect the expression level of integrin and vinculin in different colonies. First, WB assay was performed to analyze the integrin and vinculin expression in engineered colonies with low and high density at central regions (Fig. 3c). The quantitative results revealed that integrin expression level was enhanced with increasing cell density, independently of cell colony size (D-800 and D-1500) (Fig. 3d). Similarly, vinculin had the same results with integrin expression at central regions (Fig. 3e). Additionally, the expression level of integrin and vinculin at peripheral regions was obtained by WB analysis (Fig. 3f). Heterogeneous curvature at the periphery induced integrin (Fig. 3g) and vinculin (Fig. 3h) expression, especially in the colony with low density. Furthermore, talin-1 was stained to investigate the cytoskeleton–ECM or cell–cell interaction due to the adhesion-related connecting bridge action (Supplementary Fig. 2d). The staining results indicated that engineered cells would form more talin-1 in the peripheral region than in the central region, and similar results were observed with integrin and vinculin expression. Therefore, FA arrangement (integrin, vinculin, and talin-1) was regulated by cell density, curvature radius, and interfacial heterogeneity.

### Heterogeneous cytoskeleton organization to regulate YAP mechanotransduction and nuclear laminA/C remodeling on microstencils

**Fig. 4:**
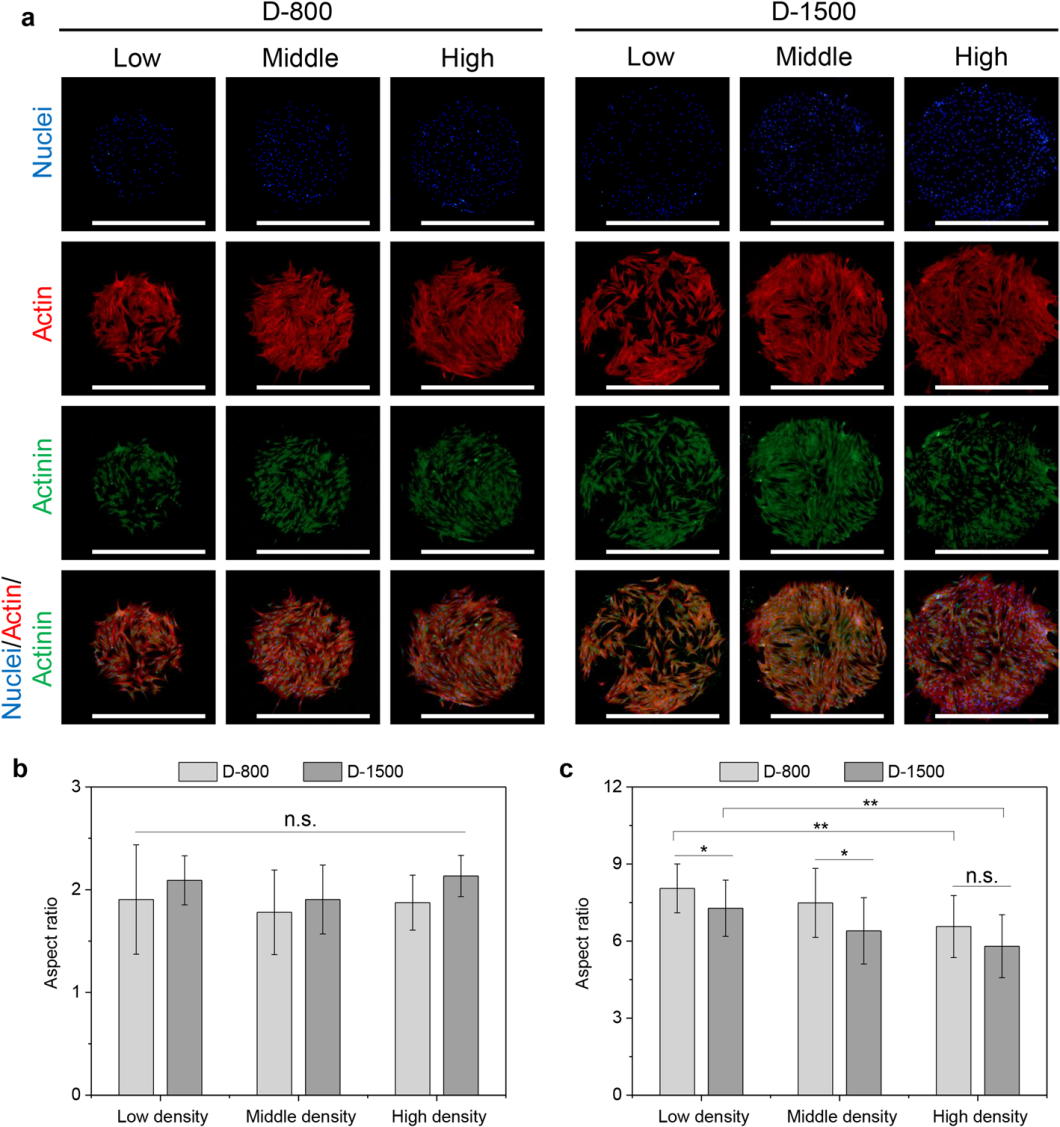
Regulation of interfacial heterogeneity on actin and actinin arrangement. **a** Fluorescent pictures of nuclei (blue), actin (red) and actinin (green), scale bar represents 1000 μm; **b** elongation (aspect ratio) of the engineered human mesenchymal stem cells (hMSCs) in the central regions; **c** elongation (aspect ratio) of the engineered hMSCs in peripheral regions, data are presented as mean ±  standard deviation (SD), *n* = 5, n.s.: no significance, *p < 0.05, **p < 0.01

It has been reported that extracellular stimuli (chemical, physical, and biological cues) can be transferred into cells by ECM, integrin, and FAs to manage cytoskeleton organization in engineered hMSCs [44]. Cellular cytoskeleton fibers of actin, actinin, and myosin play an important role in affecting cell behaviors [45–47]. To probe the relationship between cell colony (density, curvature, and heterogeneity) and cytoskeleton structures, cytoskeleton fibers were costained to observe the biased cytoskeletal heterogeneity. Actin filaments were organized at the periphery of microstencils, and further actinin, one of the actin-bonding proteins, also presented the same events with actin filaments in both peripheral and central areas (Fig. 4a). Owing to the cytoskeletal heterogeneity from density and curvature difference in the peripheral and central areas, the elongation of hMSCs was analyzed to explore cytoskeleton compliance along the heterogeneous interface of microstencils. Remarkably, little significant difference was noticed for the elongation of hMSCs at the central areas (Fig. 4b). The elongation of hMSCs demonstrated a decreased tendency with increasing cell density, while curvature radius promoted aspect ratio increase at peripheral areas (Fig. 4c). Nevertheless, peripheral cells presented longer aspect ratio (elongation) than central cells. The elongated results revealed that cell density, curvature, and heterogeneity adjusted the aspect ratio of hMSCs and cytoskeleton tension to regulate cell behaviors.

**Fig. 5:**
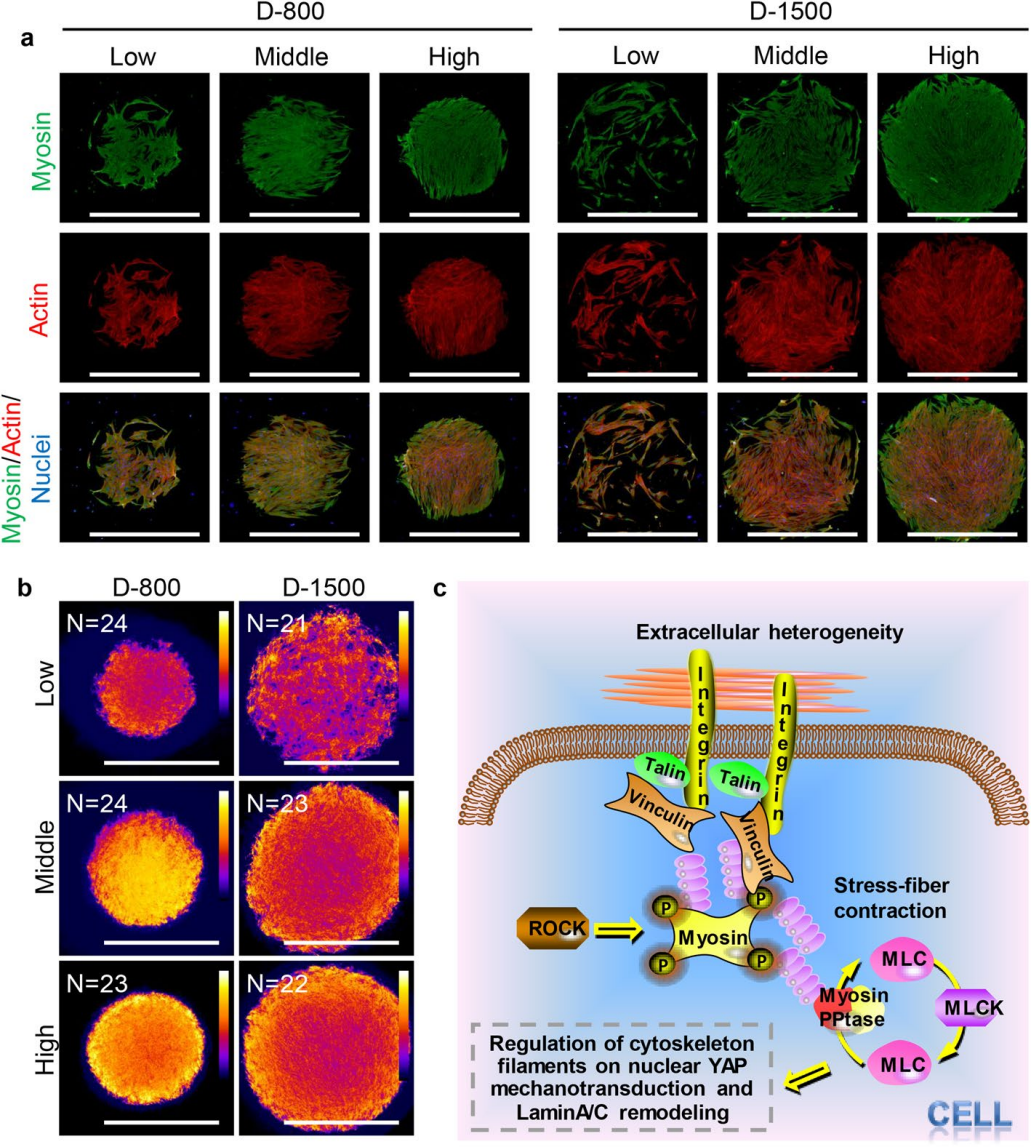
Heterogeneity of curvature-mediated myosin by the regulation of heterogeneous focal adhesion (FA) assembly in an engineered colony of human mesenchymal stem cells (hMSCs). **a** Fluorescent pictures of nuclei (blue), actin (red) and myosin (green), scale bar represents 1000 μm; **b** myosin heatmap pictures, created by overlaying more than 20 colonies into one picture, scale bar represents 1000 μm; **c** illustration of the regulation of FAs and cytoskeleton filaments on nuclear mechanotransduction; MLC: myosin light chain; MLCK: myosin light chain kinase; PPtase: phosphopantetheinyl transferase

The cytoskeleton molecule motor, myosin, is one multifunctional protein that is the driving force for cellular contractility and movement [48]. In this work, myosin was also stained to study the effect of interfacial heterogeneity on myosin distribution (Fig. 5a). The staining results showed that myosin fibers were reorganized at the periphery of microstencils, indicating correlative results with actin and actinin fibers. The reason is that myosin and actin can slide with each other to promote cell contractility on the basis of sliding filament theory. Further, the myosin heatmap was built to display that the engineered hMSCs showed an accumulation tendency of myosin at the periphery of microstencils, but not at central areas (Fig. 5b and Fig. S3). Therefore, the cytoskeleton results demonstrated that thick and thin cytoskeleton filaments did not change their length in contractility and movement, but actin filaments and the head of myosin had a series of interactions, including attachment, tilting, and detachment, to promote the relative sliding of thin filaments in cell contractility to determine cell mechanotransduction (Fig. 5c).

**Fig. 6:**
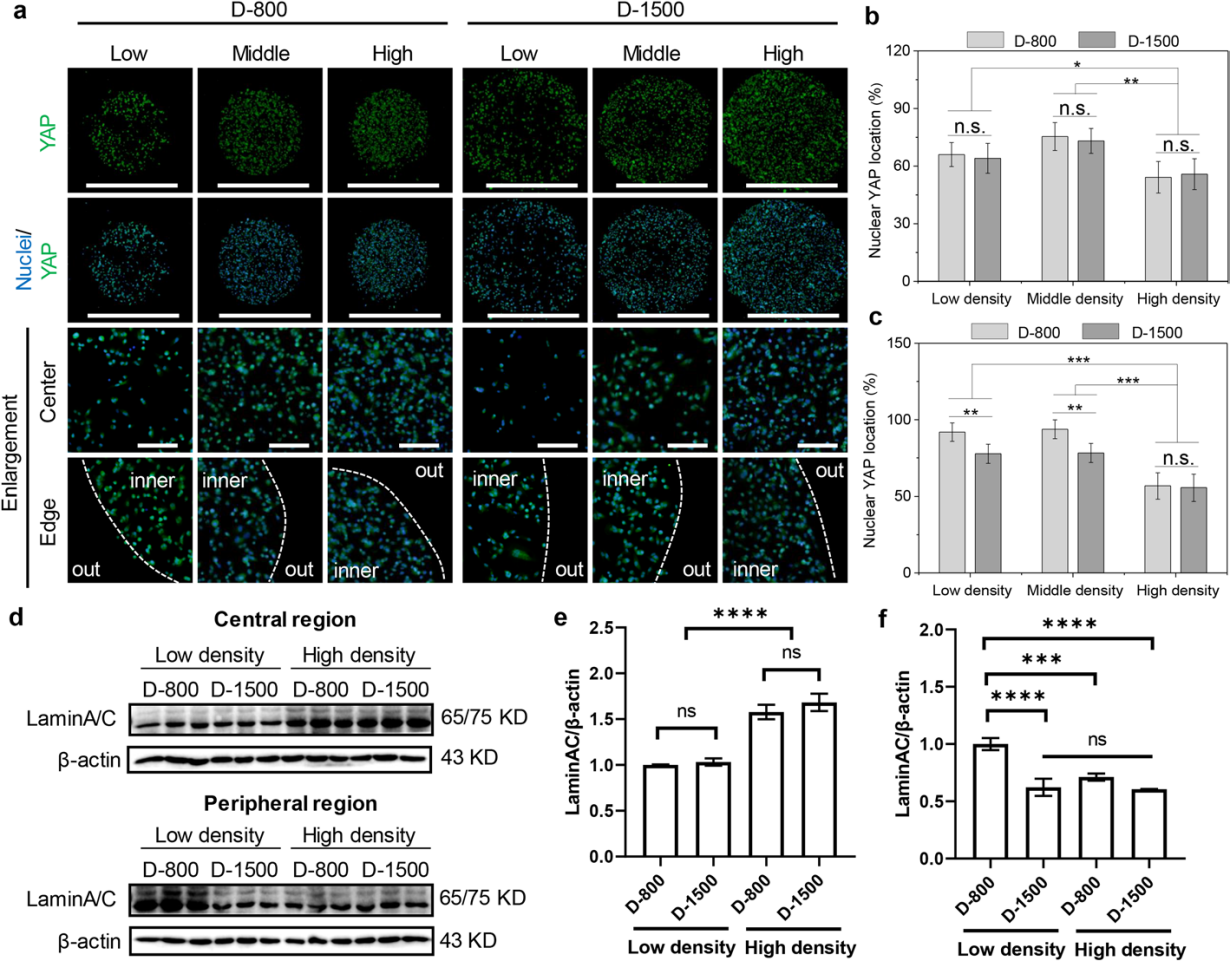
Heterogeneous focal adhesion (FA) assembly and cytoskeleton remodeling to regulate yes-associated protein (YAP) mechanotransduction and nuclear laminA/C organization in an engineered colony of hMSCs. **a** Fluorescent pictures of YAP staining (green), nuclei were stained blue, scale bar represents 1000 μm, bottom two panels are enlargement images of central and peripheral regions, scale bar represents 500 μm; **b** percentage of YAP nuclear location at the central regions; **c** percentage of YAP nuclear location at the peripheral regions, data are presented as mean ± standard deviation (SD), *n* = 5, n.s.: no significance, *p < 0.05, **p < 0.01, ***p < 0.001; **d** Western blot (WB) analysis of laminA/C in the engineered colony with low and high density; **e** quantitative laminA/C/β-actin in the central regions; **f** quantitative laminA/C/β-actin in peripheral regions, data are presented as mean ± SD, *n* = 3, n.s.: no significance, ***p < 0.001, ****p < 0.0005

According to the above-mentioned results, PDMS microstencil-mediated interfacial curvature and cell density changed the assembly of heterogeneous FA proteins and the organization of biased cytoskeletons to regulate cell functions, especially for cell nuclear mechanics [49, 50]. Herein, nuclear mechanotransduction was assessed by YAP staining to evaluate YAP nuclear translocation of heterogeneous microstencils. The YAP results demonstrated that the hMSCs displayed high YAP nuclear localization in the middle density of hMSCs at peripheral areas of microstencils (Fig. 6a). To study the interplay of interfacial heterogeneity and YAP mechanotransduction, the percentage of YAP nuclear localization was quantitatively calculated by analyzing the amount of YAP nuclear cells in whole examined cells. At central areas, the percentage of YAP nuclear localization showed the highest results in middle density, compared with low and high density (Fig. 6b). Similar results were observed with FA formation due to density-dependent cell development. Interestingly, the hMSCs at the periphery showed more YAP nuclear localization than central areas, revealing the activated Wnt and Hippo mechanical pathways in interfacial heterogeneity (Fig. 6c). In addition, the curvature was also applied to adjust YAP nuclear mechanotransduction on microstencils.

Due to the curvature-dependent heterogeneity from an engineered colony, force-sensing mechanotransduction was transferred into nuclei to change the expression of a nuclear skeleton, such as laminA/C. Therefore, the expression level of laminA/C was detected in the engineered colonies with low and high cell density by WB analysis (Fig. 6d). The laminA/C expression increased with cell density in the central regions in D-800 and D-1500 colonies (Fig. 6e). However, laminA/C was expressed more at the peripheral regions, especially for the colony with large curvature and low cell density (Fig. 6f). Further, the motor protein myosin was disturbed by 50 μM blebbistatin to reveal the crucial role of cytoskeleton structures in FA formation (integrin and vinculin) and laminA/C expression (Supplementary Fig. 4). After blebbistatin treatment, the expression levels of integrin, vinculin, and laminA/C showed the similar results in all engineered colonies, independently of cell density and curvature. The results presented that heterogeneous cytoskeleton organization could regulate YAP mechanotransduction and nuclear laminA/C remodeling by the evaluation of actin, actinin, and myosin on microstencils.

### Mechanism of curvature, spreading, and elongation on cytoskeleton and nuclear activity in the engineered hMSCs

**Fig. 7:**
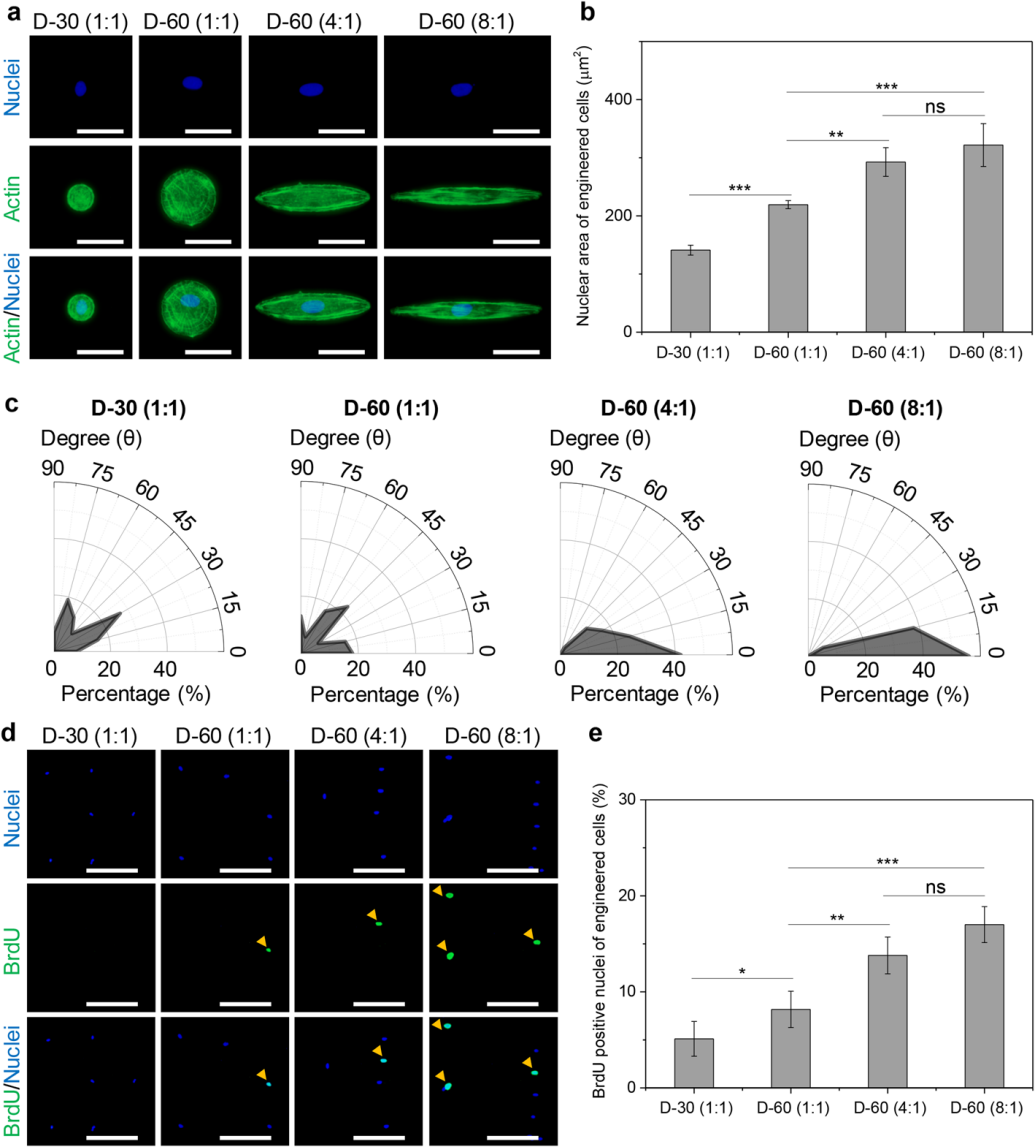
Mechanism of curvature, spreading area and aspect ratio on cytoskeleton distribution and nuclear activity in the engineered cells. **a** Fluorescent pictures of actin (green) and nuclei (blue) in the engineered cells, scale bar represents 50 μm; **b** nuclear area of the engineered cells, data are presented as mean ± standard deviation (SD), *n* = 5, n.s.: no significance, **p < 0.01, ***p < 0.001; **c** angular compliance of nuclei in the engineered cells; **d** fluorescent pictures of BrdU positive nuclei, BrdU is shown in green, nuclei in blue, scale bar represents 200 μm; **e** nuclear activity by calculating BrdU positive cells in the engineered cells, data are presented as mean ± SD, *n* = 5, n.s.: no significance, *p < 0.05, **p < 0.01, ***p < 0.001

It has been reported that cell curvature can affect cell spreading and adhesion on different surfaces, altering cytoskeletal structures and regulating cell functions, such as proliferation, migration, and metabolic balance [51–54]. To prove the influence of cell curvature, spreading, and elongation on cell activity, the engineered microcircles were designed to control the curvature, spreading, and elongation of hMSCs (Supplementary Fig. 5). Cytoskeleton structures of the engineered hMSCs were stained to analyze actin reorganization and nuclear activity (Fig. 7a). Compared with the small adhesion area of 30 μm^2^ cells, large cells formed more thick and well-organized cytoskeleton networks on 60 μm^2^ cells. Moreover, the elongated and curved cells promoted the compliance of actin filaments along with the stretched axis. The adjusted cytoskeleton would also change nuclear morphogenesis (Fig. 7b). The nuclear area was enhanced with increasing cell spreading, curvature, and aspect ratio. Further, the nuclei in the engineered cells were completely oriented along the stretched direction of the engineered cells (Fig. 7c). The engineered cells transformed the stimuli of curvature, spreading, and elongation through the regulation of cytoskeletal structures into the nuclei, thereby facilitating the regulation of DNA synthesis and cell division. BrdU staining was used to investigate nuclear DNA activity (Fig. 7d). The percentage of BrdU positive nuclei was enhanced with increasing cell spreading, curvature, and aspect ratio (Fig. 7e). Therefore, nuclear activity was associated with parallel stretch of cytoskeleton (actin, actinin, and myosin), FA kinase stimulation and zyxin relocation, YAP nuclear translocation, and nuclear DNA activity, owing to the interfacial heterogeneity and curvature of microstencils. Taken together, the mechanical stimulation derived from heterogeneous FA proteins (integrin, vinculin, and talin-1) and biased cytoskeleton structures (actin, actinin, and myosin) could change YAP nuclear mechanotransduction, nuclear DNA activity, and laminA/C nuclear remodeling in the engineered hMSCs.

## Conclusions

The PDMS-based stencils were microengineered by a straightforward punching method to accurately manipulate low-to-high cell density and interfacial curvature in clustering colonies of engineered hMSCs, especially in the peripheral region. The micropatterned colony of hMSCs exhibited similar characteristics to the engineered micropores of microstencils. The cell colony clearly developed the heterogeneous morphogenesis to regulate diverse FA production (integrin, vinculin, and talin-1) and anisotropic cytoskeleton (actin, actinin, and myosin) arrangement at the periphery. The biased mechanical stimulation from the heterogeneity of FA proteins and cytoskeleton structures was frequently activated to transmit the force-related signals in the ECM or microenvironment into cells and nuclei to supervise cellular behaviors and fates. These mechanical pathways triggered nuclear mechanotransduction by activating YAP nuclear translocation and laminA/C remodeling to accommodate interfacial changes in heterogeneous FA formation and biased cytoskeleton tension at the periphery of stencils. The results may provide insights into cell-density-dependent clustering architecture and interfacial curvature to elucidate the relationship between molecular heterogeneity and asymmetric mechanotransduction.

## Supplementary Information

Supplementary Material 1.

## Data Availability

All data generated or analyzed during this study are included in this published article and its additional files. Further details are available from the corresponding author upon request.

## Abbreviations

PDMS: Polydimethylsiloxane
PVA: Polyvinyl alcohol
hMSCs: Human mesenchymal stem cells
SD: Standard deviation
FA: Focal adhesion
DMEM: Dulbecco’s modified eagle medium
ECM: Extracellular matrix
PFA: Paraformaldehyde
YAP: Yes-associated protein
BrdU: 5-bromodeoxyuridinc
DNA: Deoxyribonucleic acid
WB: Western blot
