# Supplementary material for: Heterogeneous focal adhesion cytoskeleton nanoarchitectures from microengineered interfacial curvature to oversee nuclear remodeling and mechanotransduction of mesenchymal stem cells
Source: Cell Mol Biol Lett. 2025 Jan 24;30:10. doi: 10.1186/s11658-025-00692-z (PMC11762875; doi:10.1186/s11658-025-00692-z)
Supplement: Supplementary file 1 — Supplementary Material 1. [file 11658_2025_692_MOESM1_ESM.docx]

**Supporting Information**

**Heterogeneous FA-cytoskeleton nanoarchitectures from microengineered interfacial curvature to oversee nuclear remodeling and mechanotransduction of mesenchymal stem cells**

Huayu Fan^a,b,1^, Hui Zhao^c,1^, Yan Hou^d,1^, Danni Meng^d^, Jizong Jiang^d^, Eon-Bee Lee^e^, Yinzheng Fu^c^, Xiangdong Zhang^a,^*, Rui Chen^a,b,f,^*, Yongtao Wang^d,^*

^a^ Luoyang Orthopedic Hospital of Henan Province, Orthopedic Hospital of Henan Province, Zhengzhou 450008, Henan, China.

^b^ Graduate School, Henan University of Chinese Medicine, Zhengzhou 450046, Henan, China.

^c^ Zhengzhou Revogene Technology Co., LTD, Airport District, Zhengzhou 451162, Henan, China.

^d^ School of Medicine, Shanghai University, Shanghai 200444, China.

^e^ Department of Aquatic Life Medicine, Pukyong National University, Busan 48513, South Korea.

^f^ School of Nursing, Fujian University of Traditional Chinese Medicine, Fujian 350122, Fuzhou, China.

^1^ The authors equally contribute to this paper.

Correspondence to:

Prof. Rui Chen, E-mail: 41903878@qq.com

Prof. Xiangdong Zhang, E-mail: 1058290809@qq.com

Dr. Yongtao Wang, E-mail: yongtao_wang@shu.edu.cn


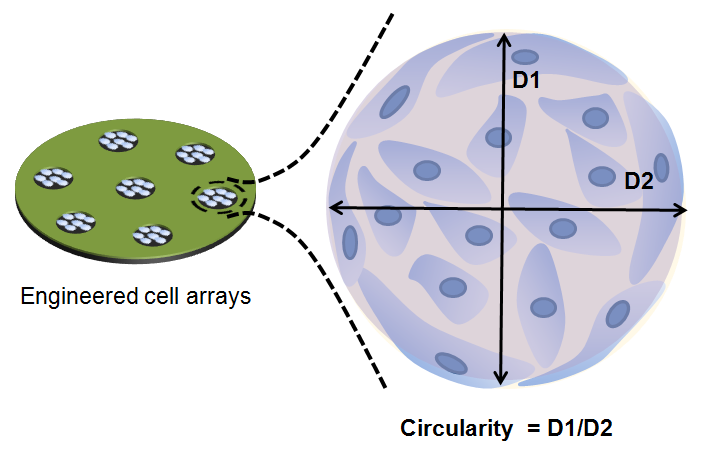


**Fig. S1**  Circularity of microengineered cells arrays. The percentage of long axis and short axis was defined as the circularity.


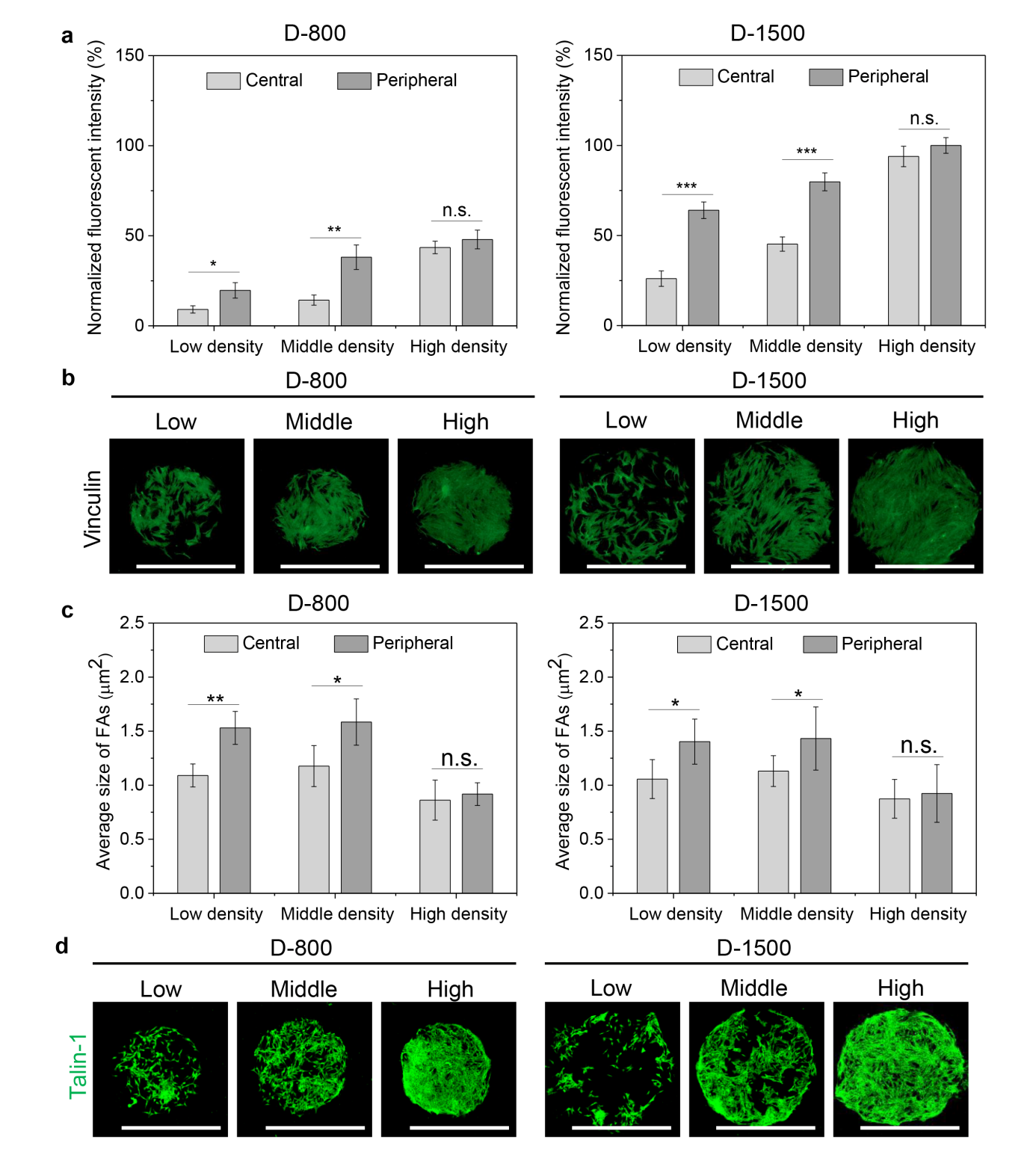


**Fig. S2**  Influence of heterogeneous curvature on Integrin, Vinculin and Talin-1 formation. (a) Normalized Integrin fluorescent intensity in D-800 and D-1500 engineered colony. (b) Fluorescent pictures of Vinculin staining (green). Scale bar: 1000 μm. (c) Average size of FAs (Vinculin) in D-800 and D-1500 engineered colony. (d) Fluorescent pictures of Talin-1 staining (green). Scale bar presents 1000 μm. Data present mean ± SD, n = 5, n.s.: no significance, **p* < 0.05, ***p* < 0.01, ****p* < 0.001.


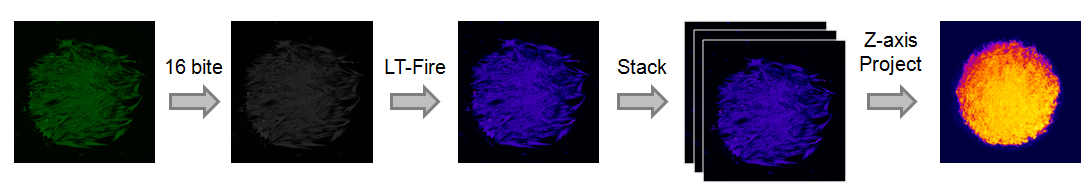


**Fig. S3**  Myosin heatmap images were formed by stacking multiple myosin images into one image after lookup table, stack and z-axis project.


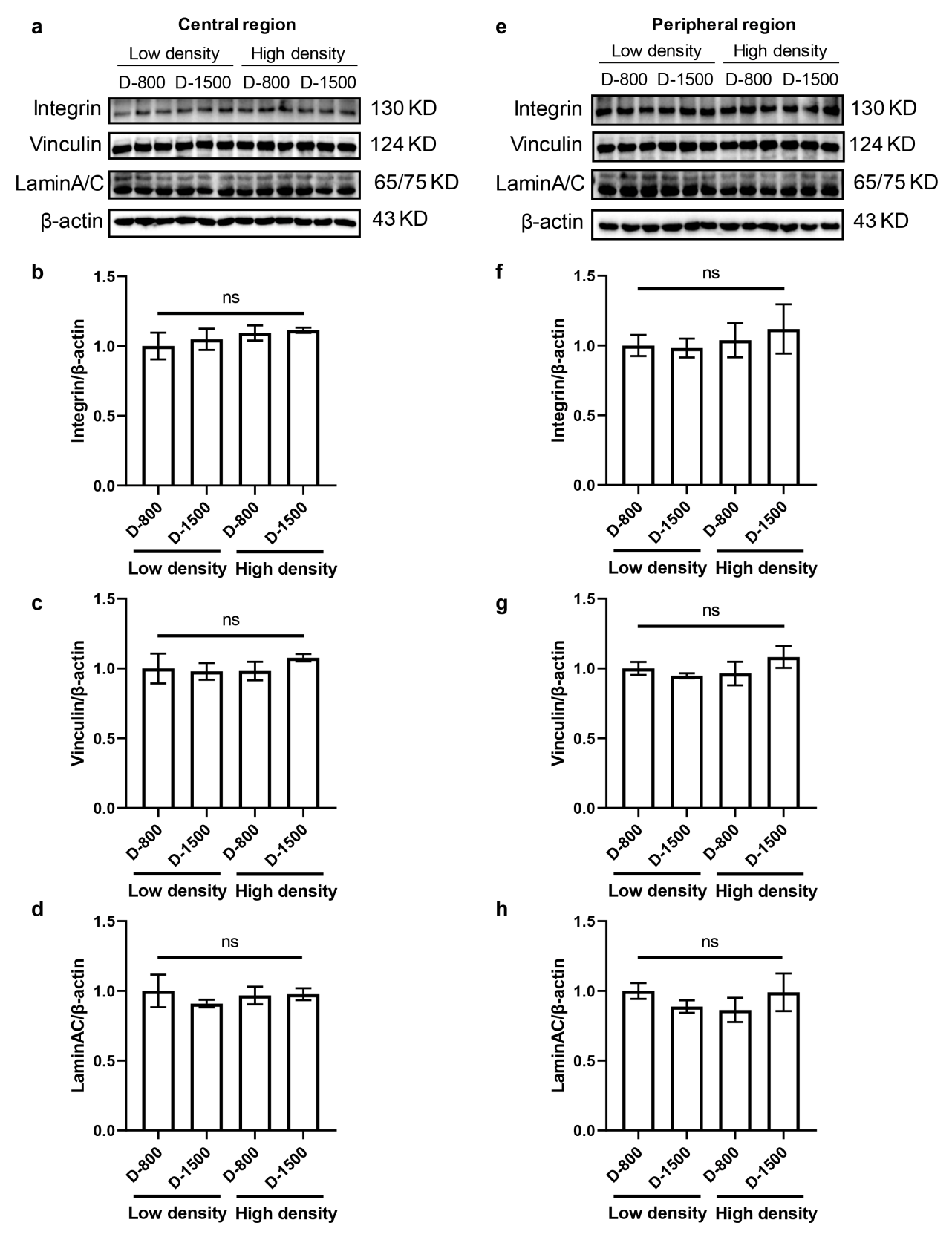


**Fig. S4**  WB analysis of Integrin, Vinculin and LaminA/C after treatment with 50 uM blebbistatin. (a) WB expression of Integrin, Vinculin and LaminA/C in the central region of engineered colony. Quantitative expression level of Integrin (b), Vinculin (c) and LaminA/C (d) after treatment with blebbistatin in the central region. (e) WB expression of Integrin, Vinculin and LaminA/C in the peripheral region of the engineered colony. Quantitative expression level of Integrin (f), Vinculin (g) and LaminA/C (h) after treatment with blebbistatin at the periphery. Data present mean ± SD, n = 3, ns: no significance.


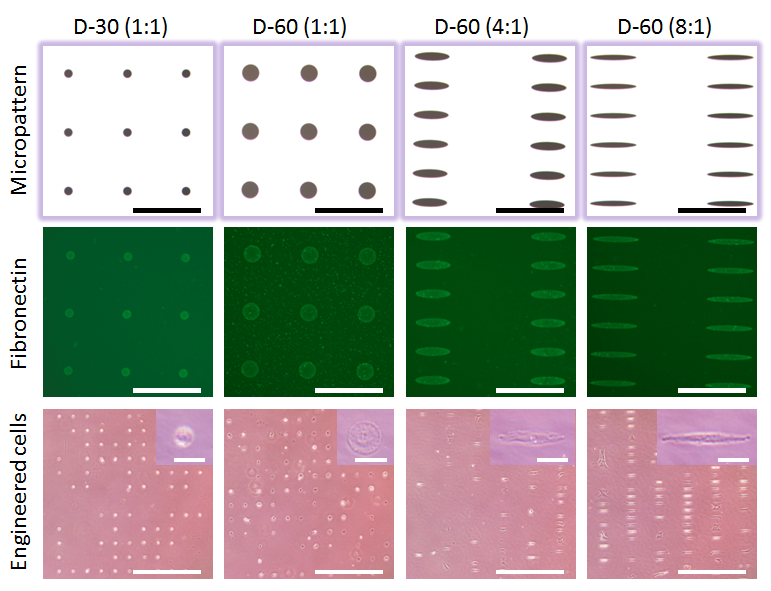


**Fig. S5** Preparation and characterization of microcircle arrays. Top panel was the prepared micropatterns. Fibronectin was coated on the microcircle arrays to enhance cell attachment. The hMSCs were seeded on the microarrays to control cell morphology. Scale bar: 200 μm. Inset images were enlarged images of cell morphology. Scale bar: 50 μm.
